# Cut-off values of serum interleukin-6 for culture-confirmed sepsis in neonates

**DOI:** 10.1038/s41390-022-02329-9

**Published:** 2022-10-10

**Authors:** Erik Küng, Lukas Unterasinger, Thomas Waldhör, Angelika Berger, Lukas Wisgrill

**Affiliations:** 1 Division of Neonatology, Pediatric Intensive Care & Neuropediatrics, Department of Pediatrics and Adolescent Medicine, Comprehensive Center for Pediatrics, Medical University of Vienna, Vienna, Austria; 2 Department of Epidemiology, Center for Public Health, Medical University of Vienna, Vienna, Austria

## Abstract

**Introduction:**

Neonatal sepsis accounts for 0.97% of all disability-adjusted life years worldwide. Interleukin-6 has been used in sepsis diagnosis, but cut-off values are missing.

**Methods:**

Neonates admitted to the neonatal wards with measurements of serum interleukin-6 born between September 2015 and September 2019 were retrospectively analysed. Mean serum interleukin-6 values of patients who never had increased laboratory parameters of infection nor died during their stay and mean interleukin-6 values on the day of blood sampling for a later positive culture in patients with culture-confirmed sepsis were analysed for each time period.

**Results:**

In all, 8.488 values in 1.695 neonates, including 752 very-preterm-infants and 701 very-low-birthweight infants, were analysed. The AUC for interleukin-6 was 0.84–0.91 in all neonates, 0.88–0.89 in very-preterm and 0.89–0.91 in very-low-birthweight infants. Using interleukin-6 cut-off values of 80 pg/ml on day of life 1, 40 pg/ml on day of life 2–7 and 30 pg/ml after day of life 7, a sensitivity of 75% and a specificity of 81% for culture-confirmed sepsis were achieved. In very-preterm infants, the corresponding values were 74% for sensitivity and 83% for specificity and in very-low-birthweight infants 74% and 86%, respectively.

**Conclusion:**

Serum interleukin-6 has high accuracy for the detection of neonatal sepsis.

**Impact:**

- Serum interleukin-6 can be used with high accuracy to detect sepsis in neonates with the cut-off values of 80 pg/ml on day of life 1, 40 pg/ml on day of life 2–7 and 30 pg/ml after day of life 7.
- Serum interleukin-6 can be used with high accuracy to detect sepsis in neonates and very-preterm as well as very-low-birthweight infants.
- Interleukin-6 values display distinct cut-off values depending on the chronological age of the infant.
- Our article provides the first cut-off values for interleukin-6 in the first days of life in neonates.

## Introduction

Neonatal sepsis accounts for 0.97% of all disability-adjusted life years worldwide,^1^ demonstrating higher percentages compared to colon and rectum cancer, asthma or breast cancer. Neonatal sepsis has a high mortality of 11–19%^2^ and is associated with brain injury^3^ as well as poor neurodevelopmental and growth outcomes in early childhood.^4^ These adverse outcomes call for early detection and rapid intervention.

Since sepsis is a systemic inflammatory response to infection, isolation of bacteria from blood is considered the gold standard.^5–7^ Due to the low obtainable blood volume in very-low-birthweight infants (VLBW infants, birthweight <1500 g) of around 0.5–1 ml,^8^ the high percentage of low-level bacteraemia of 68%^8^ and the long duration to a positive blood culture up to 61 h,^9^ several parameters have been tested for their accuracy in diagnosing neonatal sepsis including procalcitonin, lipopolysaccharide-binding protein, presepsin and interleukin-6 (IL-6).^7,10^

IL-6 is a multifunctional cytokine participating in immune response, haemopoiesis and acute-phase reactions.^11^ In response to infection, IL-6 enhances the production of IgM, IgG and IgA as well as the proliferation of helper T-cells and thereby plays a crucial role in host defence mechanisms.^11^ After exposure to bacterial endotoxins, IL-6 concentrations rise before acute-phase reactants including C-reactive protein (CRP).^10,12^ IL-6 can be determined in cord blood or serum yielding different diagnostic accuracy. Cord blood IL-6 has a sensitivity of 87–100% for early-onset sepsis.^13–15^ Serum IL-6 has a sensitivity of 75–85% and a specificity of 72.8–88% for the diagnosis of early-onset sepsis,^16,17^ and a sensitivity of 80–93.8%^10,18,19^ and a specificity of 80–96%^18–20^ for late-onset sepsis, thereby outperforming CRP.^10^ Serum IL-6 concentrations quickly fall to undetectable values during antibiotic treatment.^10,20^

The high sensitivity and negative predictive value of IL-6 make it a suitable candidate for diagnosing neonatal sepsis in combination with the high specific CRP, but reliable cut-off values for serum IL-6 in term and preterm infants are missing.

The aim of this study was to determine optimal cut-off values for serum IL-6 in diagnosing culture-confirmed sepsis in neonates and very-preterm (VP, born before 32 weeks of gestation) and VLBW infants.

## Methods

**Fig. 1:**
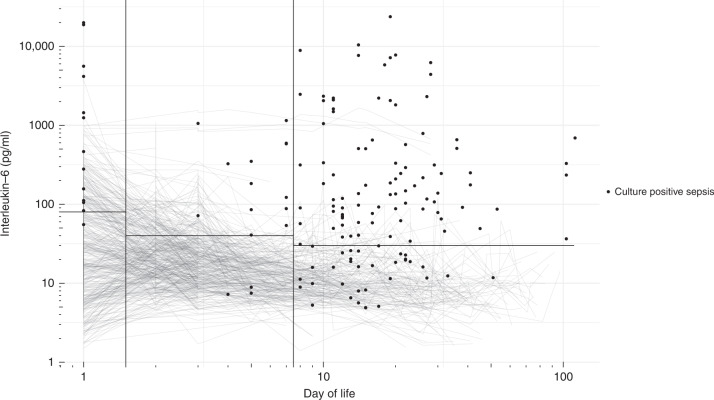
Serum interleukin-6 values of healthy neonates and on the day of culture-confirmed sepsis. Serum interleukin-6 values of neonates who never had increased laboratory parameters of infection following Neo-KISS criteria nor died during their stay at our unit (lines) and mean serum interleukin-6 values on the day of blood sampling for a later positive culture in culture-confirmed sepsis following Neo-KISS criteria (dots). Vertical lines divide the study episodes into day of life 1, day of life 2–7 and after day of life 7 and horizontal lines represent the cut-off values during the episodes with 80 pg/ml on day of life 1, 40 pg/ml on day of life 2–7 and 30 pg/ml after day of life 7.

This retrospective cohort study was approved by the ethics committee of the Medical University of Vienna (No. 2081/2019), followed the STARD^21^ and SAMPL^22^ guidelines and was conducted in accordance with the Declaration of Helsinki. All inborn neonates admitted to the neonatal wards with measurements of IL-6 born between 1 September 2015 and 1 September 2019 were analysed. Outborn patients were excluded because we could not assess the medical history with consistent accuracy compared to inborn patients. Values taken after surgery were excluded to account for immunologic responses to surgery or implants. Laboratory (Vitros 250, Ortho Clinical Diagnostics, Raritan, or Cobas 6000, Roche, Basel, Switzerland) and microbiologic parameters were routinely determined independently by the respective departments. Data were obtained from the electronic patient data management system (ICCA, Philips Healthcare, Amsterdam, Netherlands) and the microbiology database (MOMO, Medexter Healthcare, Vienna, Austria). Due to physiologic changes in serum IL-6 with postnatal age as visible in Fig. 1, time periods for cut-off determination were set as day of life (DOL) 1, DOL 2–7 and after DOL 7. Based on the continuous physiological changes in IL-6 after birth, separate analyses for early- and late-onset sepsis were not performed. To determine cut-off values, two groups were defined: healthy neonates and neonates with culture-confirmed sepsis. Healthy neonates were defined as neonates who never had measurements of increased laboratory parameters of infection nor died during their stay at our unit. Increased laboratory parameters of infection were defined following Neo-KISS criteria^23^ as a white-blood count <5 × 10^2^/µl (×1 = ×10^9^/l), immature-to-total ratio >0.2, CRP >2 mg/dl (×10 = mg/l) and a platelet count <100 × 10^2^/µl (×1 = ×10^9^/l). Neonates with culture-confirmed sepsis were defined following Neo-KISS criteria.^23^ When coagulase-negative staphylococci were detected as single pathogen in blood or cerebrospinal fluid cultures—following Neo-KISS criteria^23^—one condition of the following was required for classification as true coagulase-negative staphylococcal sepsis: white-blood count <5 × 10^2^/µl (×1 = ×10^9^/l), immature-to-total ratio >0.2, CRP >2 mg/dl (×10 = mg/l) and a platelet count <100 × 10^2^/µl (×1 = ×10^9^/l).

**Fig. 2:**
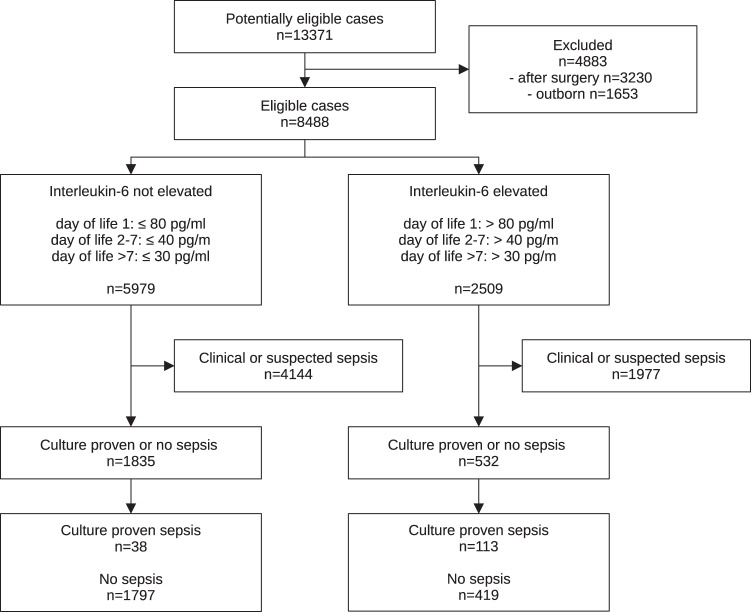
STARD flowchart. STARD flowchart with number of interleukin-6 values as cases in all neonates.

Mean serum IL-6 values per patient per time period in healthy neonates and mean serum IL-6 values per patient on the day of blood sampling for a later positive culture in neonates with culture-confirmed sepsis were analysed for cut-off determination for each time period using ROC analysis. Next, the calculated cut-off values were validated using all available IL-6 values (as seen in the STARD flowchart, Fig. 2) and sensitivity, specificity, as well as positive and negative predictive values, were calculated. IL-6 values taken in healthy infants and IL-6 values from the day of blood sampling for a later positive culture were used as reference to calculate diagnostic accuracy.

The area under the curve (AUC) and diagnostic accuracy were then calculated for each subgroup consisting of preterm (P, born before 37 weeks of gestation) infants, VP infants (infants born before 32 weeks of gestation), extremely preterm (EP, born before 28 weeks of gestation) infants, VLBW (birthweight <1500 g) infants and extremely low-birthweight (ELBW, birthweight <1000 g) infants. Calculations for AUC and diagnostic accuracy for the term population were not possible due to the low number of culture-positive cases in the term infant population (*n* = 3). Statistical analysis was performed in the ‘R’ statistic environment^24^ with the ‘pROC’ package^25^ using ‘DeLong’ method^26^ for calculating the AUC and the ‘epiR’ package^27^ using a two-by-two table with exact binomial confidence levels for calculating diagnostic accuracy. No multiple testing was performed. Alpha level was set to 0.05.

## Results

Between 1 September 2015 and 1 September 2019, 8.488 values in 1.695 neonates were analysed after exclusion (shown in Fig. 2). Of those IL-6 values, 2.216 were determined in 927 healthy neonates (referred as controls—497 in term infants and 1.719 in preterm infants), and 151 IL-6 values in 109 neonates were determined on the day of a positive culture in episodes of culture-confirmed sepsis (referred as cases—3 in term infants and 148 in preterm infants). This results in 2.367 IL-6 values in 1.036 neonates used for cut-off calculation (Supplementary Table 1) and 8.488 IL-6 values in 1.695 neonates used for diagnostic accuracy analysis. The demographic parameters are listed in Table 1.

**Table 1:** Characteristics of analysed patients.

Analysed patients	Total	Control	Cases	*p* value
Number of patients, *n* (%)	1695 (100)	927 (54.7)	109 (6.4)	–
VLBW infants, *n* (%)	701 (41.4)	214 (23.1)	97 (89)	<0.001
VP infants, *n* (%)	752 (44.4)	252 (27.2)	96 (88.1)	<0.001
PPROM, *n* (%)	517 (30.5)	278 (30)	58 (53.2)	0.001
Preterm labour, *n* (%)	552 (43.9)	256 (39.9)	74 (79.6)	<0.001
Mothers with chorioamnionitis, *n* (%)	224 (23)	90 (17.6)	35 (50.7)	<0.001
Mothers with pre-eclampsia, *n* (%)	82 (4.8)	32 (3.5)	2 (1.8)	0.56
Gestational age at birth, weeks (SD)	32.5 (5.0)	34.3 (3.9)	26.8 (3.8)	<0.001
Birthweight, g (SD)	1910 (1000)	2230 (860)	960 (530)	<0.001
Percentile at birth, mean (SD)	39.5 (28)	40.2 (27.4)	42 (26.6)	0.44
Under 10th percentile, *n* (%)	295 (17.1)	141 (15.1)	11 (9.6)	0.22
Female patients, *n* (%)	763 (45.0)	413 (44.6)	50 (45.9)	0.94
Multiples, *n* (%)	458 (27.0)	268 (28.9)	30 (27.5)	0.91
Length of stay, day (SD)	30.7 (41.5)	15.7 (23.8)	80.3 (47.5)	<0.001
Mortality, *n* (%)	104 (6.1)	0 (0)	18 (16.5)	<0.001

Numbers (*n*), percentages (%), with the mean (standard deviation) or number (percentage). Not available data were omitted for calculation and comparison. VLBW infants: very-low-birthweight infants, birthweight <1500 g; VP infants: very-preterm infants, born before 32 weeks of gestation; PPROM: preterm premature rupture of membranes.

‘Cases’ were defined as patients with episodes of culture-confirmed sepsis, ‘Controls’ were defined as healthy patients who never had an episode of infection during their hospital stay. ‘Cases’ and ‘Controls’ were used for cut-off calculation, whereas the ‘Total’ cohort was used for diagnostic accuracy analysis.

**Fig. 3:**
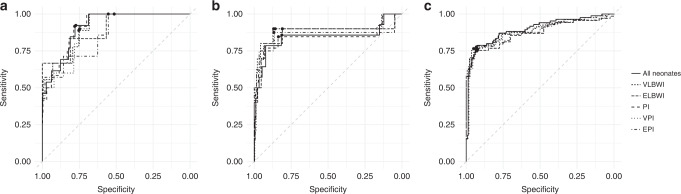
Receiver operating characteristic curves for serum interleukin-6 and culture-confirmed sepsis. Receiver operating characteristic curves for interleukin-6 in neonates, very-low-birthweight infants (VLBWI, birthweight <1500 g), extremely low-birthweight infants (ELBWI, birthweight <1000 g), preterm infants (PI, born before 37 weeks of gestation), very-preterm infants (VPI, born before 32 weeks of gestation) and extremely preterm infants (EPI, born before 28 weeks of gestation) for day of life 1 (**a**), day of life 2–7 (**b**) and after day of life 7 (**c**). The points represent the cut-off values of 80 pg/ml (**a**), 40 pg/ml (**b**) and 30 pg/ml (**c**).

The AUC for serum IL-6 was 0.84–0.91 as shown in Fig. 3. The AUC for serum IL-6 was 0.91 (95% CI: 0.85–0.97; 754 controls and 13 cases) on DOL 1, 0.84 (95% CI: 0.68–1.00; 540 controls and 14 cases) on DOL 2–7 and 0.90 (95% CI: 0.85–0.94; 232 controls and 85 cases) after DOL 7. Based on the ROC-curves shown in Fig. 3, cut-off values were set to 80 pg/ml on the DOL 1, 40 pg/ml on DOL 2–7 and 30 pg/ml after DOL 7, resulting in a sensitivity of 75% (95% CI: 67–82%), a specificity of 81% (95% CI: 79–83%), a positive predictive value of 21% (95% CI: 18–25%) and a negative predictive value of 98 (95% CI: 97–99%) as seen in Table 2.

**Table 2:** Diagnostic accuracy of serum Interleukin-6.

	Patients, *n* (%)	AUC	Sen (95% CI)	Spe (95% CI)	NPV (95% CI)	PPV (95% CI)
All infants	1695 (100)	0.84–0.91	75% (67–82%)	81% (79–83%)	98% (97–99%)	21% (18–25%)
P infants	1260 (74.3)	0.84–0.91	74% (67–81%)	83% (81–84%)	97% (96–98%)	27% (23–32%)
VP infants	752 (44.4)	0.88–0.89	74% (65–81%)	83% (81–85%)	96% (95–97%)	36% (30–41%)
EP infants	375 (22.1)	0.86–0.88	74% (65–82%)	84% (80–87%)	93% (90–95%)	54% (46–62%)
VLBW infants	701 (41.4)	0.89–0.91	74% (66–81%)	86% (84–88%)	96% (94–97%)	43% (37–50%)
ELBW infants	378 (22.3)	0.87–0.9	75% (66–81%)	82% (77–86%)	90% (86–93%)	60% (51–68%)

The diagnostic accuracy including area under the ROC-curve (AUC), sensitivity (Sen), specificity (Spe), negative predictive value (NPV) and positive predictive value (PPV) of serum interleukin-6 in all neonates, preterm (P, born before 37 weeks of gestation) infants, very-preterm (VP, born before 32 weeks of gestation) infants, extremely preterm (EP, born before 28 weeks of gestation) infants, very-low-birthweight (VLBW, birthweight <1500 g) infants and extremely low-birthweight (ELBW, birthweight <1000 g) infants in numbers and percentages.

Of the 1695 study infants, 435 were term infants and 1260 were preterm infants. The group of preterm infants included among others 752 VP infants, 375 EP infants, 701 VLBW infants and 378 ELBW infants. The AUC for serum IL-6 as well as sensitivity, specificity, and negative predictive value were similar in all groups as visible in Table 2. Interestingly, the positive predictive value was higher in EP and ELBW infants.

**Fig. 4:**
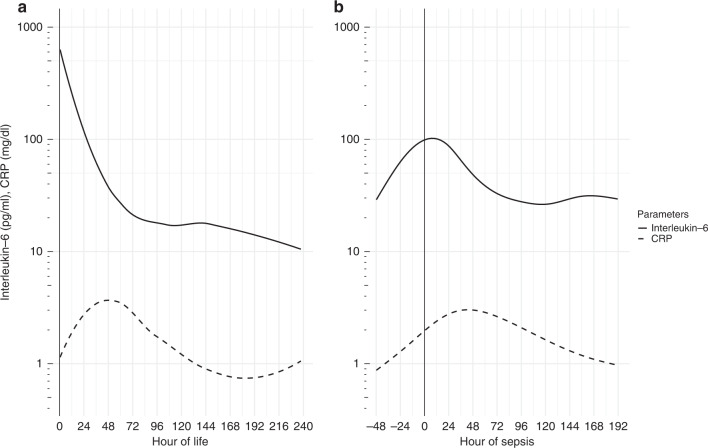
Interleukin-6 and C-reactive protein and their development over time in sepsis. Serum interleukin-6 and C-reactive protein (CRP) in culture-confirmed sepsis on day of life 1 (**a**) and after day of life 7 (**b**).

The development of IL-6 compared to CRP in patients with culture-confirmed sepsis on DOL 1 and after DOL 7 is depicted in Fig. 4. In patients with culture-confirmed sepsis on DOL 1, serum IL-6 starts high from its peak and declines until 48 h of life while CRP starts increased but needs up to 48 h to reach its peak. In patients with culture-confirmed sepsis after DOL 7, serum IL-6 has its peak on the day of a positive culture, while CRP needs 36 h to reach its peak. Serum IL-6 drops to normal values within 72 h while CRP needs up to 120 h to reach normal values. The median of mean serum IL-6 values per patient per time period is summarised in Supplementary Table 2.

The most common bacteria isolated in blood culture in episodes of culture-confirmed sepsis were coagulase-negative staphylococci (49.4%), *Escherichia coli* (21.9%) and *Klebsiella pneumoniae* (9%). When IL-6 levels in healthy infants were assessed for association with birthweight, gestational age at birth and chronological age, only chronological age was associated with a decrease in IL-6 as depicted in Fig. 1.

## Discussion

In the current study, we report for the first-time serum IL-6 cut-off values for the diagnosis of sepsis in neonates and preterm infants stratified for day of life. We demonstrate in a single-centre cohort of over 1500 neonates including over 700 VLBW infants and of those almost 400 ELBW infants, that serum IL-6 has a high accuracy for the diagnosis of culture-confirmed sepsis in this patient population. This is consistent with the literature.^7,17,28^ Tam et al. reported in their review from 2017, that IL-6 has a sensitivity of 54–84%, a specificity of 70–86%, a positive predictive value of 38–100% and a negative predictive value of 59–97% for early-onset sepsis, and a sensitivity of 44–100%, a specificity of 74–93%, a positive predictive value of 40–86% and a negative predictive value of 74–100% for late-onset sepsis.^7^ Due to physiologic changes in IL-6 postnatally (shown in Fig. 1) we did not divide between early-onset and late-onset sepsis, but rather calculated cut-off values at different days of life. Still, our results are consistent with the values published by Tam et al.^7^

Tessema et al., 2020, reported a very similar performance of serum IL-6 compared to our results with a sensitivity of 73.1%, a specificity of 80.2%, a positive predictive value of 37.6% and a negative predictive value of 94.8%.^28^ However, the authors proposed a much higher cut-off value of 313.5 pg/ml for the diagnosis of neonatal sepsis. We identified a significant physiologic change in serum IL-6 dependent on the day of life (shown in Fig. 1), therefore we calculated cut-off values dependent on the day of life. By contrast, Tessema et al. proposed one cut-off value independent of patient age. Furthermore, as healthy controls, we included only inborn patients without any prior surgery and only patients who never had increased laboratory parameters of infection following Neo-KISS criteria nor died during their stay at our unit, in order to exclude any patient with a clinical or possible infection. By contrast, Tessema et al. used CRP <10 mg/l in five serial measurements and negative blood culture and neonates who had not started antibiotic treatment before blood collection to define healthy controls. The physiologic change of IL-6 with the day of life and differences in control group definitions might cause the difference in the proposed cut-off values. The mean day of life reported by Tessema et al. was 4.4 (6.8).^28^ As seen in Fig. 1, serum IL-6 in healthy neonates decreases drastically at this age and one cut-off value cannot be used for accurate diagnosis. If we applied the proposed cut-off value of 313.5 pg/ml to our dataset, this would result in a sensitivity of 31%, a specificity of 97%, a positive predictive value of 39% and a negative predictive value of 95%.

Ebenebe et al., 2019, reported a cut-off value of 40 pg/ml of serum IL-6 in the first 72 h of life in preterm infants with a birthweight below 2000 g, resulting in a sensitivity of 75%, a specificity of 72.8%, a positive predictive value of 14% and a negative predictive value of 98%.^17^ While the methods used by Ebenebe et al. were similar to ours, several important points differed. We only used episodes of culture-confirmed sepsis and values taken in infants who never had an infection and only prior to surgery, while Ebenebe et al. used culture-confirmed sepsis and clinical sepsis defined as elevated CRP levels with clinical symptoms and controls defined by the absence of early-onset sepsis.^17^ As argued above, the use of one cut-off value for the first three days of life does not take into account the physiologically high serum IL-6 on the first day of life. In our dataset the mean serum IL-6 in healthy neonates with a birthweight below 2000 g was 78.9 (195.5) pg/ml on DOL 1. If we applied the proposed cut-off of 40 pg/ml for the first three days of life on neonates with a birthweight below 2000 g in our dataset, this would result in a sensitivity of 100%, a specificity of 73%, a positive predictive value of 6% and a negative predictive value of 100%. Hence, 94% of neonates would be treated with antibiotics unnecessarily.

Although Tessema et al. and Ebenebe et al. reported high accuracies for their cut-off values in their respective datasets, the proposed cut-off values do not take into account the physiologic changes in serum IL-6 in the first week of life and their high accuracy could not be reproduced when applied to our dataset. Our data in neonates and preterm infants show that cut-off values have to be dependent on patient age regardless of the definition of early-onset or late-onset sepsis. In conclusion, the similar diagnostic performance of IL-6 reported by Tessema et al.,^28^ Ebenebe et al.^17^ and us confirm the high accuracy of IL-6 as a diagnostic parameter, but the differences in cut-off values show the importance of considering the physiologic changes in serum IL-6. Hence we propose a compromise between accuracy and practicability: one cut-off value on DOL 1 at 80 pg/ml, one in the first week of life (DOL 2–7) at 40 pg/ml and one after the first week of life (after DOL 7) with 30 pg/ml.

As shown in Fig. 4, serum IL-6 starts highly elevated in cases of culture-confirmed sepsis on DOL 1, whereas CRP takes 48 h to reach its peak. Based on the high accuracy of serum IL-6, we conclude that IL-6 determined on DOL 1 is very helpful in the diagnosis of congenital sepsis.

As also shown in Fig. 4, serum IL-6 increases 24–48 h before CRP in episodes of culture-confirmed sepsis after DOL 7. This makes serum IL-6 the most accurate and first laboratory parameter to rise in cases of sepsis after the first week of life. Tzialla et al. reported cytokine levels to change rapidly, even before acute-phase reactants.^10^ Based on the high accuracy and especially the very high negative predictive value of serum IL-6, we conclude that serum IL-6 is very helpful in the diagnosis of sepsis in neonates and preterm infants. Furthermore, IL-6 falls rapidly after initiation of effective antibiotic therapy,^10,20^ which allows fast monitoring of antibiotic therapy and timely escalation or de-escalation of treatment.

Several characteristics known to be associated with increased risk of infection were significantly different in infants with culture-confirmed sepsis compared to healthy controls in our study, such as low birthweight, low gestational age, rates of premature rupture of membranes, preterm labour, and maternal chorioamnionitis, which has also been found in previous studies.^29,30^

The time from obtaining a blood sample to IL-6 results is highly dependent on the resources available. At our institution time from sampling to result is under 90 min including delivery to the laboratory and analysis on a 24/7 basis.

Our study is limited by its retrospective single-centre design and a selection bias in term neonates. Due to the low number of culture-confirmed sepsis episodes in term infants, we cannot provide diagnostic accuracy values for this patient population, which is another limitation of our study. Based on the retrospective nature of this study, it is not possible to analyse if infections were symptomatic or asymptomatic. This might also account for the sensitivity of 75% consistent with the published sensitivities of 73.1% by Tessema et al.^28^ and 75% by Ebenebe et al.,^17^ suggesting a methodical problem due to the retrospective design. In our institution, however, no routine IL-6 measurements are performed; therefore, every patient in this study had at least at one point a suspected infection leading the providing clinician to order a blood sample.

As a tertiary care academic centre, our institution is specialised in the care of preterm infants and high-risk neonates. Therefore, the amount of healthy term-born neonates in our dataset is limited. Due to this selection bias, we decided to set the definition of our healthy control group very strictly by only including neonates who never had increased parameters of infection nor died during their stay at our unit and compared them to neonates with culture-confirmed sepsis following Neo-KISS criteria to allow comparability and reproducibility. We compared neonates who definitely had an infection with neonates who definitely had no infection. This is the first study with this high quality of data in a large number of patients reporting cut-off values for serum IL-6.

In conclusion, serum IL-6 can be used with high accuracy to detect sepsis in neonates and preterm infants with the cut-off values of 80 pg/ml on DOL 1, 40 pg/ml on DOL 2–7 and 30 pg/ml after DOL 7.

## Supplementary information

Supplementary Tables

## Data Availability

The datasets generated during and/or analysed during the current study are available from the corresponding author upon reasonable request.
