# Supplementary material for: Cut-off values of serum interleukin-6 for culture-confirmed sepsis in neonates
Source: Pediatr Res. 2022 Oct 10;93(7):1969–74. doi: 10.1038/s41390-022-02329-9 (PMC10313515; doi:10.1038/s41390-022-02329-9)
Supplement: Supplementary file 1 — Supplementary Tables [file 41390_2022_2329_MOESM1_ESM.pdf]

Supplementary Table 1: 2x2 Table for diagnostic accuracy of serum Interleukin-6 (IL-6) cut-off values of 80 pg/ml on day of life 1, 40 pg/ml on day of life 2-7 and 30 pg/ml after day of life 7.

	IL-6 not elevated	IL-6 elevated	
No sepsis	1797	419	2216
Culture proven sepsis	38	113	151
	1835	532	2367

Supplementary Table 2: Median of mean serum interleukin-6 values per patient per time period (day of life, DOL) in patients who never had increased laboratory parameters of infection (control) and median of mean serum interleukin-6 values per patient on the day of blood sampling for a later positive culture in neonates with culture confirmed sepsis (sepsis) per time period in all neonates, preterm (P, born before 37 weeks of gestation) infants, very preterm (VP, born before 32 weeks of gestation) infants, extremely preterm (EP, born before 28 weeks of gestation) infants very low-birthweight (VLBW, birthweight < 1500g) infants, extremely low-birthweight (ELBW, birthweight < 1000g) infants.

	DOL 1		DOL 2-7		DOL >7	
Group	control	sepsis	control	sepsis	control	sepsis
All infants	23	465	18	153	10	95
P infants	20	372	17	183	10	96
VP infants	21	762	17	224	11	95
EP infants	68	1245	19	454	12	97
VLBW infants	22	1245	16	224	11	94
ELBW infants	77	1344	20	224	13	94
